# Effect of Mineral Admixtures on Physical, Mechanical, and Microstructural Properties of Flue Gas Desulfurization Gypsum-Based Self-Leveling Mortar

**DOI:** 10.3390/ma17102227

**Published:** 2024-05-09

**Authors:** Shiyu Wang, Yanxin Chen, Wei Zhao, Chang Chen

**Affiliations:** College of Materials Science and Engineering, Xi’an University of Architecture & Technology, Xi’an 710055, China; wangshiyu@xauat.edu.cn (S.W.); zhaowei@xauat.edu.cn (W.Z.)

**Keywords:** flue gas desulfurization gypsum-based self-leveling mortar, steel slag, silica fume, fly ash, properties

## Abstract

The production of flue gas desulfurization gypsum poses a serious threat to the environment. Thus, utilizing gypsum-based self-leveling mortar (GSLM) stands out as a promising and effective approach to address the issue. β-hemihydrate gypsum, cement, polycarboxylate superplasticizer, hydroxypropyl methyl cellulose ether (HPMC), retarder, and defoamer were used to prepare GSLM. The impact of mineral admixtures (steel slag (SS), silica fume (SF), and fly ash (FA)) on the physical, mechanical, and microstructural properties of GSLM was examined through hydration heat, X-ray diffractometry (XRD), Raman spectroscopy, and scanning electron microscopy (SEM) analyses. The GSLM benchmark mix ratio was determined as follows: 94% of desulfurization building gypsum, 6% of cement, 0.638% each of water reducer and retarder, 0.085% each of HPMC and defoamer (calculated additive ratio relative to gypsum), and 0.54 water-to-cement ratio. Although the initial fluidity decreased in the GSLM slurry with silica fume, there was minimal change in 30 min fluidity. Notably, at an SS content of 16%, the GSLM exhibited optimal flexural strength (6.6 MPa) and compressive strength (20.4 MPa). Hydration heat, XRD, and Raman analyses revealed that a small portion of SS actively participated in the hydration reaction, while the remaining SS served as a filler.

## 1. Introduction

In recent years, the global production of flue gas desulfurization gypsum (FGDG) has surged, with annual emissions exceeding 255 million tons [1]. Currently, with the continuous increase in energy consumption and increasingly stringent environmental requirements, the production of FGDG in China has exceeded 100 million tons [2]. The prolonged and extensive storage of FGDG not only consumes land resources, but the gypsum also contains impurities that seriously pollute the environment through water, air, and soil contamination [3,4]. Currently, FGDG is primarily utilized in the realm of building materials [5,6,7,8,9,10,11]. The use of FGDG for the remediation of phosphorous leaching in soil plants could be advantageous to improve agriculture land harvesting yields [12].

Within the domain of building construction, essential projects include the laying and backfilling of underfloor heating, as well as the leveling of floors, creating a substantial demand for self-leveling materials. Nevertheless, traditional cement-based self-leveling mortar has disadvantages such as poor thermal conductivity and low construction efficiency [13]. Gypsum, with its early strength and minimal shrinkage, proves advantageous, prompting its substitution for cement in the preparation of self-leveling materials. Canbaz et al. [14] studied the impact of various fine aggregates and water-reducing agents on the performance of gypsum-based self-leveling mortar (GSLM). The GSLM demonstrated 28-day compressive strength, shear strength, and water absorption rate of 34 MPa, 4.6 MPa, and 1.3%, respectively.

GSLMs are widely employed as fillers for floor heating systems, known for their excellent leveling performance, rapid solidification, high early strength, and versatility [15,16,17,18,19,20,21]. However, gypsum, the main component, demands a significant amount of water, leading to compromised strength in the later stages. To enhance its performance, additional materials must be incorporated. Furthermore, the need for additives to meet fluidity and setting time requirements is crucial. However, impurities in FGDG can impact additive effectiveness, limiting practical engineering use [22,23,24]. Yang [25] and Zhang [26] delved into the effect of gypsum on cement-based self-leveling mortar, highlighting its role as both a filler and a participant in hydration. Xiao et al. [27] utilized β-hemihydrate gypsum and sulfoaluminate cement in GSLM preparation, resulting in a well-connected structure and robust mechanical properties for products derived from desulfurization building gypsum.

The introduction of fillers or additives can enhance GSLM performance. Zhang et al. [28] explored the impact of aggregates on GSLM physical properties, revealing that molybdenum tailings with <45% content can serve as suitable aggregates. External additives exhibit interactive effects, with polycarboxylate-based, high-efficiency, water-reducing agents proving adept at dispersing gypsum particles [29,30].

Scholars have extensively researched the reinforcing effect of mineral admixtures on composite materials. Li et al. [31] added cement, fly ash, and lime to gypsum and observed that the compressive strength increased when the ratio of cement, fly ash, and lime was 10%, 20%, and 14.86%, respectively. Guo et al. [32] determined that a 75% desulfurization gypsum, 5% cement, and 20% mineral powder ratio yielded FGDG blocks with good physical properties. Zhou et al. [33] discovered that stone-based composite materials exhibited robust mechanical properties when aluminate cement, mineral powder, and quicklime were added to building desulfurization gypsum. However, research on mineral admixture application in self-leveling systems is limited, and mechanistic exploration is lacking.

This study employed FGDG and ordinary Portland cement as primary cementitious materials to formulate GSLM. The influence of steel slag (SS), silica fume (SF), and fly ash (FA) as mineral admixtures on the physical, mechanical, and microscopic properties of GSLM was systematically investigated, leading to the determination of optimal mineral admixture proportions. The use of various mineral admixtures effectively improves the performance of gypsum-based self-leveling mortar while significantly reducing the production cost of the material, resulting in the resourceful utilization of multiple solid wastes.

## 2. Materials and Methods

### 2.1. Materials

**Figure 1:**
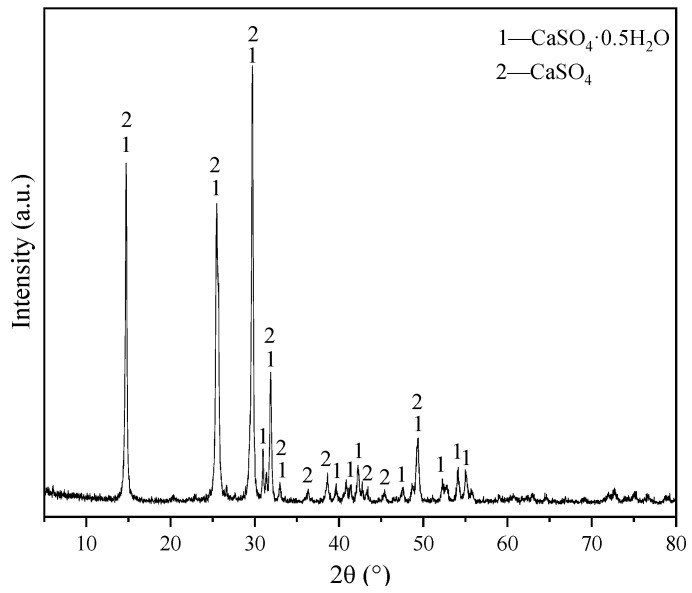
XRD spectrum of desulfurization building gypsum.

Ordinary Portland cement (Grade 42.5R) was procured from Wuxi Tianshan Cement Co., Ltd. (Wuxi, China) FGDG (CaSO_4_·2H_2_O) was obtained from Datang Power Plant (Henan, China). Its initial and final setting times were 168 and 202 min respectively, 3-day and 28-day flexural strength was 6.2 and 9.3 MPa, 3-day and 28-day compressive strength was 36.2 and 62.3 MPa, respectively. Desulfurization building gypsum underwent production at 170–190 °C, followed by aging before use. The performance characteristics of desulfurization building gypsum are detailed in Table 1 and Figure 1. As shown in the figure, the main mineral phases of desulfurization building gypsum are hemihydrate gypsum and a small amount of anhydrous gypsum. The chemical composition is shown in Table 2. Polycarboxylate superplasticizer (bulk density 500–700 g/L, containing active ingredients > 90%) can reduce the water required for mixing without reducing fluidity, Hydroxypropyl methyl cellulose ether (HPMC, active ingredient content ≥ 90%, pH 6–8) can enhance the water retention performance of mortar and prevent mortar bleeding. Polymeric amino acid retarder (bulk density 640 ± 50 g/L, fineness ≤ 300 μm) can prolong the hydration time of desulfurization gypsum, and defoamer powder polyether (pH 6–8) can reduce air bubbles in mortar. Four types of additives were sourced from Jiangsu Zhaojia Building Materials Technology Co., Ltd. (Jiangsu, China).

The chemical compositions of the three mineral admixtures—SS, SF, and FA—are presented in Table 2. SS primarily consists of CaO, SiO_2_, and Fe_2_O_3_, with an average particle size of 9.34 μm. According to the requirements of GB/T 20491-2017, “Steel slag powder used for cement and concrete”, the activity index of steel slag was tested, and its strength activity indexes at 7 and 28 days were 78.3% and 83.1%, respectively. SF is primarily composed of SiO_2_ and CaO with an average particle size of 1.45 μm. According to the requirements of GB/T 27690-2023. “Silica fume for cement mortar and concrete”, the activity index of silica fume was tested, and its strength activity index at 7 days was 70%. FA is primarily composed of SiO_2_ and Al_2_O_3_ with an average particle size of 12.94 μm. According to the requirements of GB/T 1596-2017, “Fly ash used for cement and concrete”, the activity index of fly ash was tested, and its strength activity indexes were 73.6% and 74.6% at 7 days and 28 days, respectively [34,35,36].

### 2.2. Experimental Methods

(1) Proportion of desulfurization gypsum-based self-leveling mortar

This experiment aims to prepare desulfurization gypsum based self-leveling mortar so that its performance meets the JC/T 1023-2021, “Gypsum-based self-leveling compound for floor” [37]. Four single-factor experiments were designed to determine the cement content, water-reducing agent, retarder, and methyl hydroxypropyl cellulose ether, and ultimately obtain the mix ratio of desulfurization gypsum–based self–leveling mortar.

(2) Single addition experiment of mineral admixtures

The corresponding steel slag, silica fume, and fly ash were weighed according to 4%, 8%, 12%, 16%, 20%, 24%, 28%, and 32% of desulfurization building gypsum, and separately addedto the desulfurization gypsum–based self–leveling mortar mentioned earlier for flowability and strength testing.

**Figure 2:**
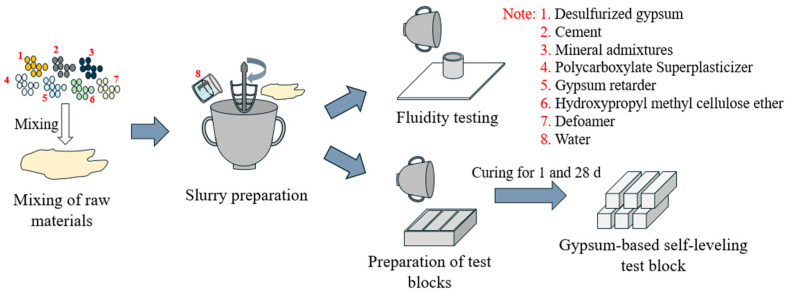
Preparation process of gypsum-based self-leveling mortar.

(3) Figure 2 depicts the preparation process of GSLM. Initially, desulfurization building gypsum, cement, mineral admixtures, and admixtures were proportionally weighed. Mineral admixtures and admixtures were added at different percentages of gypsum content, and all measured powders were introduced into the mixer for thorough blending. Proportionally weighed water was poured into the mixing pot, and the uniformly mixed powder was added to the water within a 5-second timeframe. Subsequently, the slurry was mixed for 1 min before being poured into the cement sand mold for molding.

### 2.3. Material Characterizations

The fluidity tests were conducted following JC/T 1023-2021, “Gypsum-based self-leveling compound for floor.” The fluidity test mold, with a diameter of 30 mm and height of 50 mm, was positioned horizontally at the center of the plate glass. Fresh mortar was then poured into the fluidity test mold, allowing for a 4 min free flow. The initial fluidity was determined by taking the arithmetic mean value of its diameters in both vertical directions. For the 30 min fluidity, the mortar stood for 30 min initially, and its fluidity was measured after 30 s of stirring.

Mechanical strength testing was carried out using a microcomputer-controlled electronic pressure testing machine (DYE-300S, Wuxi Dejiayi Testing Instrument Co., Ltd., Wuxi, China). The loading rates were 0.05 and 2.4 kN/s, respectively, for the flexural and compressive strength. Six specimens, each having dimensions of 40 mm × 40 mm × 160 mm, were prepared for each experiment and demolded after 1 day. Three samples were taken to measure the 1-day strength. After curing for 28 days under standard conditions, the remaining samples were dried in the oven at 40 ± 2 °C for 2 days until a constant mass was achieved. Subsequently, the 28-day absolute dry strengths of the specimens were measured.

An isothermal conduction calorimeter (Calmetrix I-Cal 8000 HPC) was employed to test the hydration heat evolution of pastes after 24 h at 25 °C. X-ray diffraction (XRD, Bruker D8 Advance, Rheinstetten, Germany) was utilized to study the hydration product of GSLM pastes. The XRD tests were conducted at 40 kV, 30 mA, 4°/min, and a range of 5° to 80° with steps of 0.02°, using a CuKα anode. A Raman spectrometer (Horiba LabRAM HR Evolution, Kyoto, Japan) was used to obtain images of different molecular vibrations emitted and analyze molecular structures. The wavelength of the excitation laser was 514 nm, with a grating of 400 lines/mm, an aperture of 25 µm, an exposure time of 1 s, and 100 exposures. GSLM specimens were coated with gold, and the microstructures were analyzed by scanning electron microscopy (SEM, Zeiss Sigma 300, Oberkochen, Germany). The electron acceleration voltage of the scanning electron microscope is 20 kV.

## 3. Results and Discussion

### 3.1. Determination of the Basic Mix Ratio of Flue Gas Desulfurization Gypsum-Based Self-Leveling Mortar

**Figure 3:**
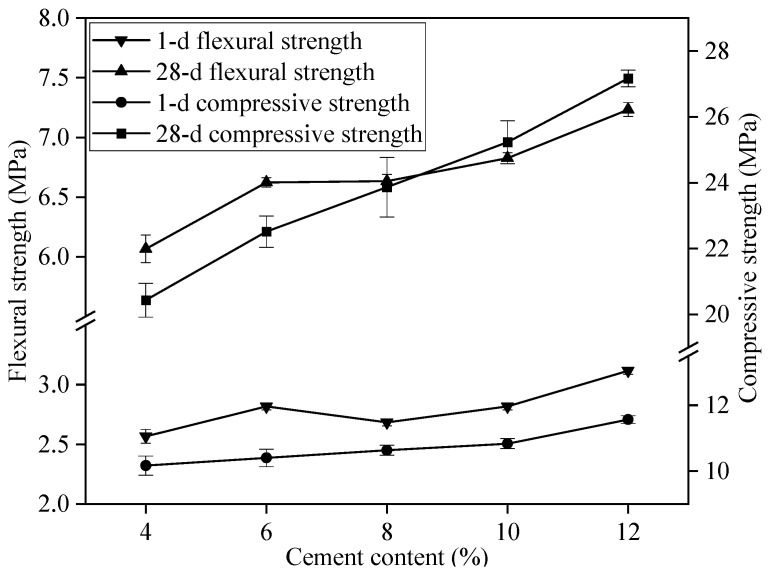
Flexural and compressive strengths of GSLMs with different cement content.

Figure 3 illustrates the flexural and compressive strengths of GSLM samples with varying cement content (the proportion of cement to total cementitious materials). The strengths exhibited an upward trend as the cement content increased. At a cement content of 12%, the flexural and compressive strengths reached their peak values, measuring 7.2 MPa and 27.2 MPa, respectively. This enhancement was attributed to the hydration reactions among the tricalcium silicate, dicalcium silicate, tricalcium aluminate, and tetracalcium aluminoferrite present in Portland cement clinker and water. These reactions led to the formation of C–S–H and needle-shaped ettringite (AFt) with cementitious properties. Consequently, the hydrated calcium silicate and AFt covered the surface of gypsum particles, filling voids in the mortar and creating a denser mortar structure, thereby enhancing the mechanical properties of GSLMs [38,39,40,41].

Within the 6% to 8% cement content range, the flexural strength remained constant at 6.6 MPa, while the compressive strength ranged from 22 MPa to 23 MPa. Despite a slight increase in compressive strength, both mechanical properties met the required standards (JC/T 1023-2021: “Gypsum-based self-leveling compound for floor”) and the specific values are shown in Table 3. Considering the substantial cost difference between cement and gypsum, it was advisable to minimize the cement content in GSLM formulation based on cost considerations. Consequently, the optimal cement content for subsequent studies was determined to be 6%.

**Figure 4:**
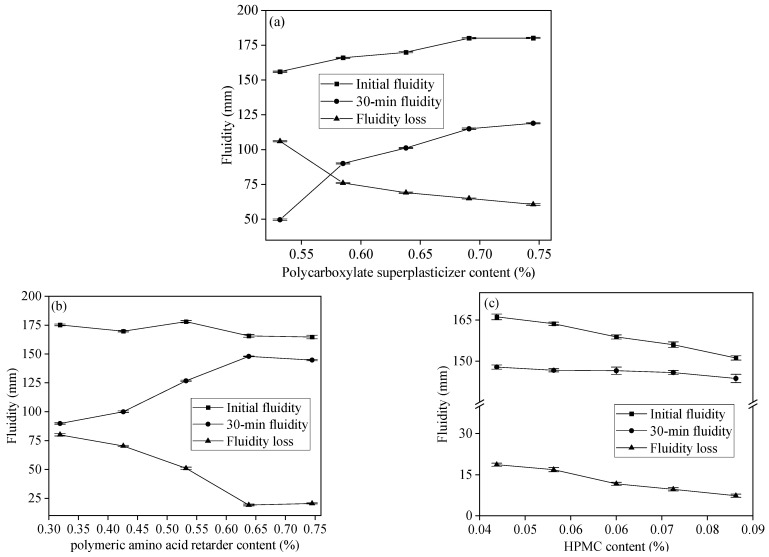
Initial fluidity, 30 min fluidity, and fluidity loss of GSLM slurries with different admixture content. (**a**) Polycarboxylate superplasticizer; (**b**) polymeric amino acid retarder; (**c**) HPMC.

Figure 4 illustrates the initial fluidity, 30 min fluidity, and fluidity loss of GSLM slurries with various admixtures (calculated additive ratio relative to gypsum). In Figure 4a, the impact of polycarboxylate superplasticizer content on the fluidity of the GSLM slurry was examined. Both initial and 30 min fluidity increased with higher polycarboxylate superplasticizer content, while fluidity loss decreased. Beyond a 0.638% polycarboxylate superplasticizer content, the fluidity of the slurry exhibited minimal change. Therefore, a 0.638% addition of polycarboxylate superplasticizer was deemed optimal.

Figure 4b shows that the initial fluidity slightly decreased, 30 min fluidity initially increased and then decreased, and fluidity loss gradually decreased with an increasing content of protein polymeric amino acid retarder. At 0.638% retarder content, the slurry demonstrated an initial fluidity of 167 mm, 30 min fluidity of 148 mm, and fluidity loss of 19 mm. Beyond 0.638% retarder content, the slurry’s fluidity showed marginal variations. Hence, 0.638% polymeric amino acid retarder was considered optimal. Furthermore, Figure 4c reveals a significant decrease in slurry fluidity with increasing HPMC content. At 0.085% HPMC content, the initial fluidity essentially met the requirements, falling within the specified range of 145 ± 5 mm (according to JC/T 1023-2021: “Gypsum-based self-leveling compound for floor”).

Based on the abovementioned results, the mixing ratios of raw materials, along with the physical and mechanical properties of the GSLM samples, are detailed in Table 3. The fluidity, flexural strength, and compressive strength of the GSLMs all satisfied the standards outlined in the GSLM standard (JC/T 1023-2021).

### 3.2. Effect of Mineral Admixtures on Physical and Mechanical Properties of Flue Gas Desulfurization Gypsum-Based Self-Leveling Mortar

**Figure 5:**
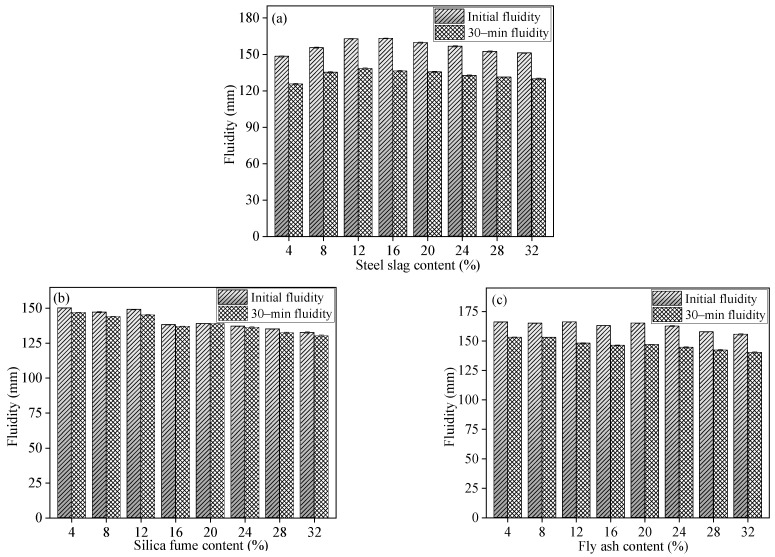
Initial and 30 min fluidity of GSLMs with different steel slag (SS), silica fume (SF), and fly ash (FA) content, respectively. (**a**) GSLM with different SS content; (**b**) GSLM with different SF content; (**c**) GSLM with different FA content.

The impact of SS, SF, and FA content on the initial and 30 min fluidity of the GSLMs is depicted in Figure 5. Both the initial and 30 min fluidity of the GSLMs initially increased and then decreased with rising SS content, as shown in Figure 5a. The 30 min fluidity fell below 140 mm when SS content increased. In Figure 5b,c, the fluidity of the slurry decreases with increasing SF and FA content. Once the content of SF and FA exceeded 16%, the 30 min fluidity of GSLM with SF remained at approximately 139 mm, while that of GSLM with FA exceeded 140 mm. Since SS and FA had particle sizes of 9.34 μm and 12.94 μm, respectively, and SF had a particle size of 1.45 μm, the addition of SS and FA had a comparable effect on initial fluidity of GSLM. However, SF’s small particles and high water adsorption capacity diminished slurry fluidity. Moreover, the 30 min fluidity of the slurries exhibited minimal change, as both SF and FA consisted of amorphous spherical particles with a glass microbead effect. Conversely, SS, with its irregular particles, increased frictional forces between particles, resulting in reduced slurry fluidity with higher SS content [24,41].

**Figure 6:**
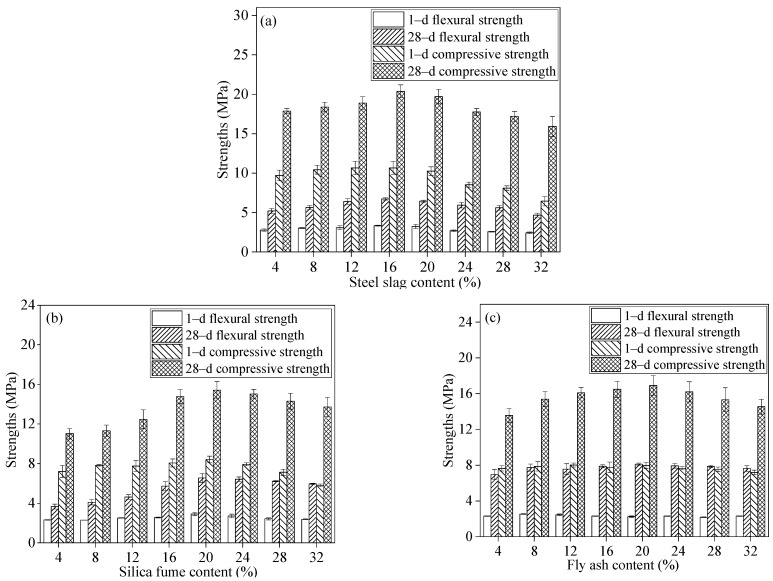
Flexural and compressive strengths of GSLMs with different steel slag (SS), silica fume (SF), and fly ash (FA) content, respectively (**a**) GSLM with different SS content; (**b**) GSLM with different SF content; (**c**) GSLM with different FA content.

Figure 6 illustrates the impact of three mineral admixture contents (SS, SF, and FA) on the mechanical properties of GSLMs. Both flexural and compressive strengths of GSLMs initially increased and then decreased with the rising content of SS, SF, and FA. In Figure 6a, the optimal flexural and compressive strengths, at 6.60 MPa and 20.40 MPa, were achieved with 16% SS content. For GSLM with SF (Figure 6b), the highest flexural and compressive strengths, measuring 6.57 MPa and 15.20 MPa, respectively, were observed at 20% SF content. Furthermore, the 1-day flexural and compressive strengths of the GSLMs with FA were approximately 2.30 MPa and 7.80 MPa, respectively (Figure 6c). However, for a 28-day curing period, the highest flexural and compressive strengths were attained with 20% FA content, measuring 8.13 MPa and 16.92 MPa, respectively.

The particles of the three mineral admixtures effectively filled the internal pores of GSLM, enhancing its compactness and leading to a continued increase in flexural and compressive strengths. Nevertheless, when the SS content exceeded 16% or the SF and FA content exceeded 20%, a downward trend in flexural and compressive strengths was observed. This suggests that excessive addition of mineral admixtures may compromise the structure between hydration products. Therefore, the optimal content for SS, SF, and FA in GSLMs was determined to be 16%, 20%, and 20%, respectively.

### 3.3. Effect of Mineral Admixtures on Microstructural Properties of Flue Gas Desulfurization Gypsum-Based Self-Leveling Mortar

**Figure 7:**
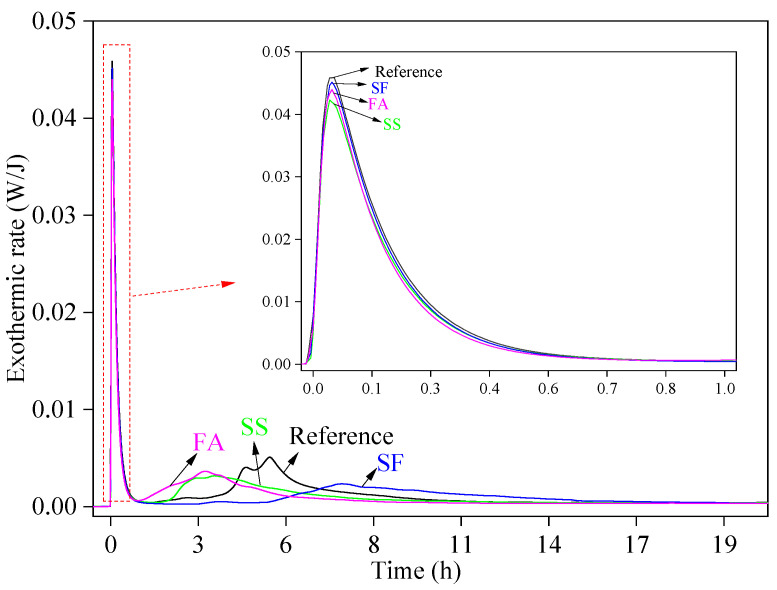
Hydration heat curves of the reference sample and GSLMs with steel slag (SS), silica fume (SF), and fly ash (FA), respectively.

Figure 7 illustrates the hydration heat release curves of the reference sample (GSLMs without mineral admixtures) and GSLMs with various mineral admixtures. The hydration processes for these samples were categorized into two stages. In the first stage, the hydration exothermic peak emerged at 0–1 h, corresponding to the hydration process of hemihydrate gypsum. The addition of mineral admixtures slightly reduced the intensity of the exothermic peaks by delaying the contact between gypsum and water [41]. However, the smaller particles of SF compared to SS and FA provided more sites for the generation of gypsum hydration products [42]. Consequently, the peak heat release rate of GSLM with SF exceeded that of GSLM with SS and FA. Despite the relatively insignificant difference in particle size between FA and SS, the amount of FA added was slightly higher than that of SS.

In the second hydration process stage, the hydration exothermic peak appeared between 1 and 16 h, corresponding to the hydration process of ordinary Portland cement (grade 42.5R) and mineral admixtures. The difference in the appearance time of the second hydration heat release peak indicated that the addition of SS and FA accelerated the hydration time, leading to a slight reduction in hydration heat release compared to the reference sample. Conversely, SF delayed the hydration time and significantly reduced heat release during hydration.

**Figure 8:**
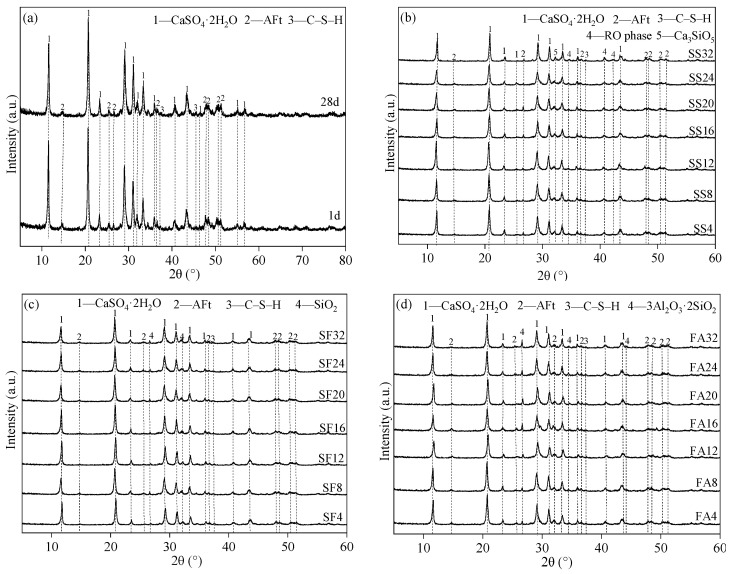
XRD patterns of the reference sample and GSLMs with different steel slag (SS), silica fume (SF), and fly ash (FA) content, respectively. (**a**) Reference sample; (**b**) GSLM with different SS content; (**c**) GSLM with different SF content; (**d**) GSLM with different FA content.

Figure 8 displays the XRD patterns of the reference sample and GSLMs with varying SS, SF, and FA content. In Figure 8a, the primary phases for the 1-day and 28-day reference sample were CaSO_4_·2H_2_O, C–S–H gel, and AFt. CaSO_4_·2H_2_O resulted from the reaction of 0.5CaSO_4_·2H_2_O with water, while C–S–H gel and AFt were products of cement hydration. There was minimal change in the content of these three hydration products between the 1-day and 28-day GSLMs, suggesting that the main hydration reactions were completed within 1 day, consistent with the results of hydration time of the reference sample.

Figure 8b illustrates the main phases—CaSO_4_·2H_2_O, C–S–H gel, AFt, RO phase, and Ca_3_SiO_5_—for the 28-day GSLM with SS. RO phase and Ca_3_SiO_5_ were present in the raw materials of SS [42,43,44,45]. The diffraction intensity of RO phase and Ca_3_SiO_5_ increased gradually with rising SS content, while the AFt content slightly increased. The AFt content reached its maximum at 16% SS content and remained constant beyond that point. Hence, with 16% SS content, the GSLM exhibited its highest strength, indicating that a portion of SS participated in hydration reactions while the remainder acted as filler.

Figure 8c,d shows the main phases: CaSO_4_·2H_2_O, C–S–H, AFt, and SiO_2_ for GSLM with SF, and CaSO_4_·2H_2_O, C–S–H, AFt, and 3Al_2_O_3_·2SiO_2_ for GSLM with FA. With increasing SF and FA content, the diffraction intensity of SiO_2_ and 3Al_2_O_3_·2SiO_2_ gradually increased. No new phases were generated during the hydration process, indicating that SF and FA functioned as fillers. The 28-day flexural and compressive strengths of GSLM with SS exceeded those of GSLMs with SF and FA.

**Figure 9:**
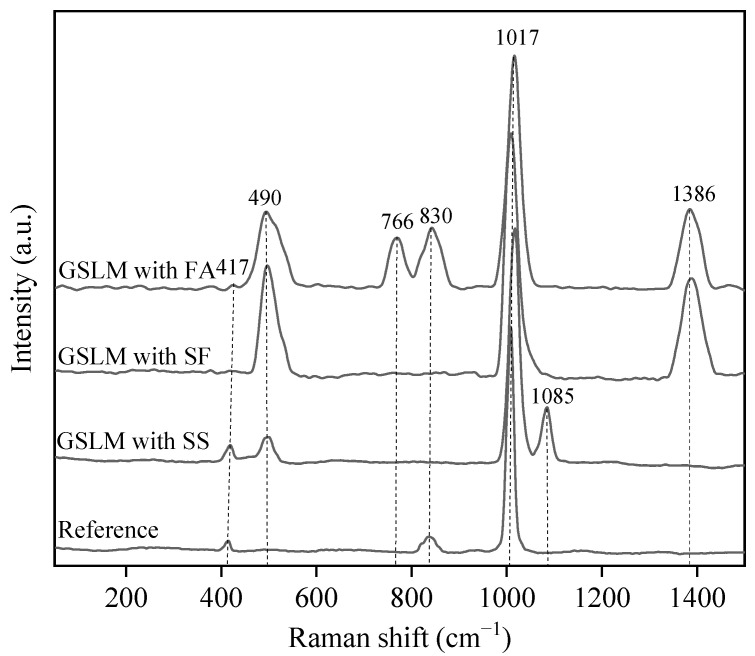
Raman spectroscopy of the reference sample and GSLMs with 16% steel slag, 20% silica fume, and 20% fly ash, respectively.

Figure 9 illustrates the Raman spectroscopic characterization of the reference sample and GSLMs with 16% SS, 20% SF, and 20% FA, respectively. The characteristic peak at 417 cm^–1^ corresponds to the symmetric bending vibration of CaSO_4_. The hydration reaction is represented by Equation (1).

CaSO_4_·0.5H_2_O + 1.5H_2_O → CaSO_4_·2H_2_O (1)

The distinct characteristic peak at 1017 cm^–1^ originated from the symmetric stretching vibration of [SO_4_^2–^]. In this context, the [SO_4_^2–^] in CaSO_4_ and AFt overlapped, leading to a higher peak height compared to the other peaks. The hydration reaction of AFt is represented by Equations (2)–(5).

C_3_A + H_2_O → C–A–H + CH (2)

C–A–H + SO_4_^2–^ → AFt (3)

C_3_S + H_2_O → C–S–H + CH (4)

C_2_S + H_2_O → C–S–H + CH (5)

Here, C–A–H represents calcium aluminate hydrate gel. Additionally, the GSLM containing SS displayed a peak at 490 cm^–1^ attributed to Si–O–Si vibration and a peak at 1085 cm^–1^ arising from the symmetric bending vibration of [SO_4_^2–^] in AFt. These findings indicated that SS reacted with water to form AFt, which was consistent with the XRD results. On the other hand, the curves of GSLM containing SF and FA all exhibited peaks at 490 cm^–1^ due to the tetrahedral vibration of amorphous [SiO_4_]^4–^ and 1386 cm^–1^ attributed to amorphous carbon vibration. Furthermore, the curve of GSLM containing FA showed peaks at 760 and 830 cm^–1^, reflecting tetrahedral vibration of [SiO_4_]^4–^. These observations suggest that SF and FA did not participate in hydration, corroborating the XRD results.

**Figure 10:**
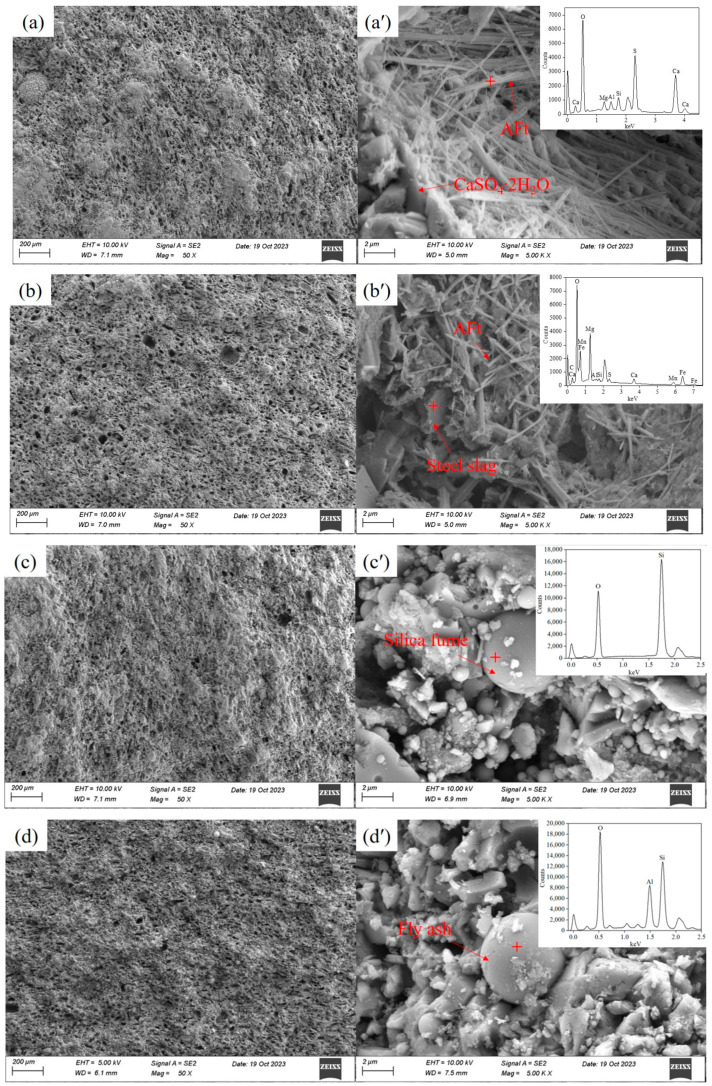
SEM morphology of the reference sample and GSLMs with 16% steel slag, 20% silica fume, and 20% fly ash, respectively. (**a**–**d**) is the cross-section of the reference sample and GSLMs with 16% steel slag, 20% silica fume, and 20% fly ash, respectively, (**a’**–**d’**) is a locally enlarged view of the cross-section of the reference sample and GSLMs with 16% steel slag, 20% silica fume, and 20% fly ash, respectively.

Figure 10 illustrates the microstructures of the reference sample and GSLMs with 16% SS, 20% SF, and 20% FA. In Figure 10a–d, the similar pore structures of the specimen section imply that these samples shared pore structures determined by the hydration products of gypsum. Furthermore, in Figure 10a, sheet-like and needle-shaped dihydrate calcium sulfate crystals were interwoven and irregularly distributed. The C–S–H gel produced by cement hydration, along with a small amount of acicular AFt, filled the pores of calcium sulfate dihydrate crystals, contributing to a more compact structure and improved 28-day strength of GSLM.

Figure 10b–d reveal more AFt crystals in GSLM with SS than GSLM with SF and FA, indicating the partial participation of SS in the hydration reaction. Additionally, AFt crystals generated from GSLM with SS were slightly thicker and shorter than those in the reference sample, causing minor damage to the connection between hydration products. Consequently, the 28-day flexural and compressive strengths of GSLM with SS were marginally lower than those of the reference sample. Smooth circular SF and FA particles, filling the pores of hydration products and not participating in hydration reactions, are prevalent in Figure 10c,d. The weak interfaces between the particles and hydration products, owing to the smooth particle surface, made them prone to crack propagation under stress. Therefore, the 28-day flexural and compressive strengths of GSLM with SF and FA were lower, which was consistent with the strength results.

SF and FA, with their regular circular shapes and microbead effect, enhanced GSLM slurry fluidity. In contrast, the irregular shape of steel slag particles increased friction force in the GSLM slurry. Consequently, GSLM slurry with SF and FA experienced less fluidity loss after 30 min, consistent with previous fluidity test results.

## 4. Conclusions

(1) The GSLM with SF exhibited the lowest initial fluidity, attributed to the finer particles, larger specific surface area, and higher water absorption capacity of SF particles. However, the smooth spherical nature of SF, with a glass bead effect, had minimal impact on the 30 min fluidity of GSLM.
(2) The strength of GSLM demonstrated an initial increase followed by a decrease with rising SS, SF, and FA content. The mineral admixtures effectively filled internal pores in GSLM, but excessive content could compromise the structure between hydration products. Consequently, the optimal content for SS, SF, and FA in GSLMs is 16%, 20%, and 20%, respectively.
(3) The hydration products of GSLMs with SS, SF, and FA mainly comprised CaSO_4_·2H_2_O, C–S–H gel and AFt. A small amount of SS exhibited continued hydration due to the stimulation of calcium hydroxide. The spherical nature of SF and FA, combined with a weak interface with hydration products, facilitated crack propagation from the interface under stress.
(4) In the future, the effects of three-phase composition, particle size of desulfurization building gypsum, and multiple mineral admixtures on the performance of gypsum-based self-leveling mortar should be researched.

## Figures and Tables

**Table 1:** Composition and performance of desulfurization building gypsum.

Project	Numerical Value
Anhydrite (%)	15.98
Hemihydrate gypsum (%)	84.02
Dihydrate gypsum (%)	0.00
Setting time (min)	3
2-h compressive strength (MPa)	4.06
2-h flexural strength (MPa)	2.26
Requirement of normal consistency (g/100 g)	85

**Table 2:** Chemical compositions of the raw materials (%).

Compositions	Na_2_O	MgO	Al_2_O_3_	SiO_2_	SO_3_	CaO	TiO_2_	K_2_O	MnO	Fe_2_O_3_	Other
Gypsum	-	0.81	1.07	1.97	54.10	40.60	-	0.20	-	0.44	0.81
Cement (OPC)	-	0.64	4.21	18.59	4.22	65.95	0.23	-	-	2.95	3.21
Steel slag	0.27	7.78	4.39	15.30	0.38	39.20	1.39	-	4.93	22.70	3.66
Silica fume	-	1.34	0.12	45.43	0.05	52.63	-	0.03	0.02	0.22	0.16
Fly ash	1.07	1.19	24.30	50.60	1.52	8.50	1.27	2.16	-	8.60	0.79

**Table 3:** Mix proportion and performance of gypsum-based self-leveling materials.

Project	Numerical Value	Requirements in JC/T 1023-2021
Desulfurization building gypsum (%)	94	-
Portland cement (%)	6	-
Polycarboxylate superplasticizer (%)	0.638	-
Polymeric amino acid retarder (%)	0.638	-
Defoamer (%)	0.085	-
HPMC (%)	0.085	-
Water-to-cement ratio	0.54	-
Initial fluidity (mm)	150	-
30 min fluidity (mm)	145	≥140
24-h flexural strength (MPa) strength/MPa	2.8	≥2.0
24-h compressive strength (MPa)	10.5	≥5.0
28-day flexural strength (MPa) strength/MPa	6.6	≥6.0
28-day compressive strength (MPa)	22.5	≥20.0

## Data Availability

Data are contained within the article.
